# Posterior Injured Vertebra Column Resection and Spinal Shortening for Thoracolumbar Fracture Associated with Severe Spinal Cord Injury: A Retrospective Case-Control Observational Study

**DOI:** 10.1155/2022/9000122

**Published:** 2022-10-06

**Authors:** Zhiyue Shi, Yingsong Wang, Jingming Xie, Jin Zhou, Tingbiao Zhu, Tao Li, Zhi Zhao, Ying Zhang, Ni Bi, Quan Li

**Affiliations:** Departments of Orthopaedic Surgery, 2nd Affiliated Hospital of Kunming Medical University, 374# Dianmian Road, Kunming 650101, Yunnan, China

## Abstract

**Background:**

Thoracolumbar spinal fracture associated with severe spinal cord injury (sSCI) is a kind of severe traumatic spine injury. Although various approaches are currently used to treat sSCI-related thoracolumbar fractures, the neurological function of patients has not been significantly improved by surgery.

**Objective:**

To evaluate the therapeutic effects of the new procedure of posterior injured vertebra column resection (PIVCR) and spinal shortening for the treatment of thoracolumbar fracture associated with sSCI.

**Methods:**

In this retrospective case-control observational study, we included 66 patients with thoracolumbar fractures associated with sSCI in our institution from January 2015 to December 2017. According to the different surgical approaches, the patients were allocated to group A (*n* = 32, received simple posterior decompression and fixation) and group B (*n* = 34, received PIVCR and spinal shortening). All patients' clinical and radiologic outcomes were collected to evaluate retrospectively. The clinical outcomes were gathered, including the intraoperative blood loss, operative time, visual analog scale (VAS) score, and American Spinal Injury Association (ASIA) impairment scale. The radiologic outcomes were collected involving the range of spinal shortening, canal encroachment, heights of the anterior edge of the vertebral body, and the Cobb angle.

**Results:**

There was no significant difference in the two groups regarding preoperative demographic data, VAS scores, segmental kyphosis Cobb, canal encroachment, and neurological status. The range of spinal shortening in group B was an average 1.57 ± 0.40 cm and 36.45 ± 6.56% of the height of the single spinal motion segment. Due to the characteristics of the surgical procedure, group B got complete decompression of the spinal cord and better postoperative canal decompression than group A. Thus, better clinical outcomes, including neurological improvement, loss of corrective Cobb angle, and VAS improvement, were shown in group B at the follow-up investigation than those in group A (*P* < 0.05).

**Conclusion:**

PIVCR and spinal shortening surgical procedure is a safe, reliable, and effective approach to treating thoracolumbar fracture associated with sSCI.

## 1. Introduction

According to incomplete estimates, the prevalence of SCI worldwide (spinal cord injury) is 223 to 755 people per million inhabitants, and the incidence of SCI per million inhabitants per year is about 10.4% to 195.4% [1, 2]. Severe spinal cord injury (sSCI) severely burdens society and families. A large portion of patients who suffer from traumatic spine injuries is mainly located in the thoracolumbar junction, which could bring about the occurrence of neurological deficits with a rate of 22%-45% [3]. In order to alleviate the neural compression, correct segmental kyphosis, rebuild vertebral body height, construct and stabilize the spine, and enhance neurological recovery, therapeutic methods including surgery (anterior, posterior, and anterior combined with posterior approaches) and pharmacological therapy are adopted to treat thoracolumbar fracture associated with sSCI [4–8]. However, neurological improvement is not always ideal by the treatments above, especially for those with complete neurological deficits. According to the research, the rate of neurological improvement for patients with traumatic sSCI in thoracic and thoracolumbar is only 22.4%, and merely 7.7% of patients with complete sSCI could get neurological improvement [9]. With the Frankel scale in thoracolumbar fracture, patients with incomplete sSCI could acquire postoperative neurological function improvement by an average of 1.3 grade [10, 11].

Surgical decompression early within 72 hours is found to significantly influence the potential for neurological improvement in patients with thoracolumbar spine injury [12]. Some studies have shown that high interspinal pressure in the injured spinal cord, spinal cord blood flow (SCBF) reduction, and interspinal circumstance disorder are critical factors that could affect the improvement of sSCI [13–16]. Effective methods urgently need to be adopted to improve the neurological deficit for thoracolumbar fractures. Posterior injured vertebra column resection (PIVCR) and spinal shortening could decompress the spinal cord circumferentially, reduce the tension of the spinal cord, improve spinal cord perfusion, and improve neurological deficit [17–25]. We applied PIVCR and spinal shortening to treat thoracolumbar fractures associated with sSCI and achieved significant neurological improvement. Therefore, it is necessary to study the clinical efficacy of PIVCR and spinal shortening in thoracolumbar fractures associated with sSCI.

To the best of our knowledge, this is the first retrospective study to report the utilization of PIVCR and spinal shortening for treating patients with thoracolumbar fractures associated with sSCI. This retrospective case-control observational study evaluated clinical effects by comparing the new approach (PIVCR and spinal shortening) and simple posterior decompression and fixation. The prime objective of this study is to determine whether the patients with thoracolumbar fracture associated with sSCI could get better clinical outcomes and neurological improvement with the surgery of PIVCR and spinal shortening than those with simple posterior decompression and fixation. The secondary objective of this study is to assess the safe range of spinal shortening in the thoracolumbar spine.

## 2. Methods

### 2.1. Ethical Approval

This study received ethical approval from the Ethics Committee at the 2nd affiliated Hospital of Kunming Medical University. Due to the retrospective characteristic, written informed consent was not applicable for this study.

### 2.2. Study Design and Patients

This retrospective case-control observational study was performed at the 2nd affiliated Hospital of Kunming Medical University. We included patients with thoracolumbar fractures associated with sSCI who received surgery in our institution from January 2015 to December 2017.

The inclusion criteria for eligible patients are as follows: (1) without complete transection of the spinal cord in the injury segment at preoperative magnetic resonance imaging (MRI); (2) severe neurological function impairment graded as A/B/C according to American Spinal Injury Association (ASIA) impairment scale; (3) single injured vertebral segment located at T10 to L1; and (4) TLICS scores ≥5 points. Patients with serious osteoporosis or without follow-up results were excluded from this study.

From January 2015 to December 2016, a total of 32 patients received simple posterior decompression and fixation and were assigned to group A as a control group. From January 2016 to December 2017, 34 patients received PIVCR and spinal shortening and were allocated to group B. All patients underwent surgery performed by the same medical team.

### 2.3. Surgical Procedures

#### 2.3.1. Simple Posterior Decompression and Fixation in Group A

**Figure 1:**
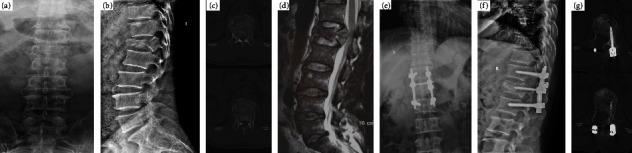
A 42-year-old male patient who was suffering from severe neurological deficit with a grade of ASIA B on admission. Preoperative X-ray (a, b) show L1 fracture with Cobb angel 22°, CT (c) with severe spinal canal encroachment, MRI (d) showed that the spinal cord was not transection in the injury segment. Undergoing simple posterior decompression fixation, patient achieved to correct segmental kyphosis deformity, restore vertebral body height (e, f), direct good decompress the neural elements (g) and neurological function improved to ASIA D.

Under general anesthesia, a posterior midline incision was made. Pedicle screws were then implanted two or three levels by freehand technique. The fracture reduction was achieved by distraction from the screw and rod on both sides. Then, hemilaminectomy or laminectomy was performed on the side of the spinal cord with severe compression and the posterior aspect of the dural sac was decompressed. The dural sac was gently retracted and protected with a nerve retractor. The fragments of fracture in front of the dural sac were meticulously removed with a curette or pushed into vertebral body with a curette to receive further decompression. The spinal reconstruction was achieved by filling autogenous bone into the injured vertebra or filling a titanium mesh cage with autogenous bone into the injured vertebra segment. During operation, methylprednisolone (30 mg/kg) was used to protect the spinal cord before spinal canal decompression (Figure 1).

#### 2.3.2. One-Stage Posterior Injured Vertebral Column Resection and Spine Shortening in Group B

**Figure 2:**
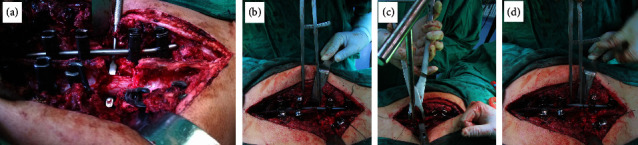
PIVCR and spinal shortening treated for T12 fracture associated with SCI. (a) shows posterior injured vertebral column resection; (b, c, d) show the spinal shortening procedure.

**Figure 3:**
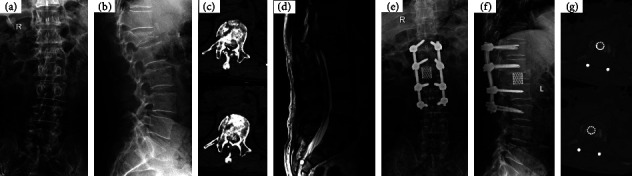
A 48-year-old male patient who was suffering from severe neurological deficit with a grade of ASIA A on admission. Preoperative X-ray (a, b) show L1 fracture with Cobb angel 19°, CT (c) with severe spinal canal encroachment, MRI (d) showed that the spinal cord was not transection in the injury segment. Undergoing PIVCR and spinal shortening, patient achieved to correct segmental kyphosis deformity, restore vertebral body height (e, f), direct good decompression the neural elements (g) and neurological function improved to ASIA E.

Under general anesthesia, patients were placed in the prone position, and posterior midline incision was made. Pedicle screws including two or three levels upper and lower of the injured vertebral body were then implanted by freehand technique, respectively. The injured vertebral body did not need to be implanted. Through the costotransverse approach, the costotransverse joints and 2-3 cm of bilateral ribs in the thoracic segment were excised, respectively, and then the lateral wall of the vertebral body was exposed easily under meticulous subperiosteal dissection on each side. The spinous process, bilateral lamina, and complete facet joint of the injured level needed to be removed. Then, the dural sac and nerve roots were clearly exposed and the posterior aspect of the dural sac was decompressed. Dural sac and nerve root were then gently retracted and protected with a nerve retractor. The fragments of the fracture in front of the dural sac were meticulously pushed into the vertebral body with a curette to receive decompression and spinal cord safety. The pedicle on one side was removed. The vertebral body was removed with a curette or rongeur and upper and lower intervertebral discs were removed. A temporary rod was placed when one side of the osteotomy was completed to keep the spinal stability. Subsequently, the same surgical process was performed on the opposite side. After the whole vertebral body as well as the upper and lower intervertebral discs were removed, the spinal cord was completely 360° decompressed. The spinal column was divided into two free segments into the cephalic and caudal sides, which were only connected by the spinal cord. The segmental kyphosis and spinal dislocation were easily got correction; meanwhile, safely controllable spine shortening was achieved (Figure 2). The principle of spine shortening was that the dural sacs should be not obvious shrinking and buckling. The spinal cord tension was reduced than preshortening through direct palpation on the dural sacs. Then, the permanent fixation rods were placed. If the dural sac was injured by accident, the repair was performed to reduce the risk of postoperative cerebrospinal fluid leakage. After accomplishing the process of spinal shortening, the height of the anterior and the resection space was measured. A titanium mesh filled with autograft bone was positioned in the midline of the inferior endplate of the cephalad vertebra and the superior endplate of the caudal vertebra from lateral side. The compression of the spine achieved the stability of the titanium mesh. Finally, a drain was placed, and the muscle, fascia, and skin were closed in sequence. During the operation, methylprednisolone (30 mg/kg) was used to protect neurological function 5 min before spinal canal decompression (Figure 3).

### 2.4. Clinical Outcomes and Radiologic Evaluation

The criteria were gathered to evaluate the clinical outcomes, including the intraoperative blood loss, operative time, VAS score, and ASIA impairment scale. The ASIA impairment scale assessed sensory and motor levels which were affected by the spinal cord injury and the VAS score assessed neurological outcomes and back pain.

**Figure 4:**
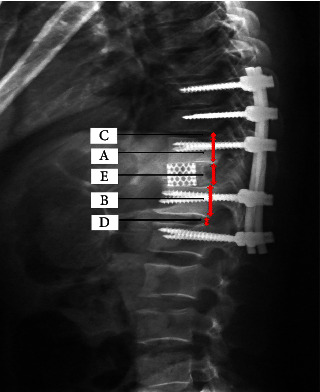
Range of spinal shortening and percentage of spinal shortening of single spinal motion segment (total height of posterior edge of vertebral body + cephalad disc + caudal disc) were calculated using the following equation on the postoperative lateral radiograph. Range of Spinal shortening: *X* = (*A* + *B*)/2 + *C* + *D* − *E*, percentage of spinal shortening of single spinal motion segments: *Y* = *X*/[(*A* + *B*)/2 + *C* + *D*] × 100%, where A is the height of posterior edge of cephalad vertebral body, B is the height of posterior edge of caudal vertebral body C is the height of posterior edge of the cephalad disc, D is the height of posterior edge of caudal disc, and E is the height of posterior edge of titanium mesh.

Cobb angle, canal encroachment, heights of the posterior edge of the vertebral body, and range of spinal shortening were measured as the radiologic outcomes. Traumatic segment kyphosis was measured by Cobb angle between the upper and lower end plates of the adjacent vertebrae of the fracture on the lateral radiograph. Radiologic assessment of spinal canal encroachment rate was calculated on computed tomography. As Lin et al. [11] demonstrated, the height of the posterior edge of the vertebral body was calculated as follows: the restore rate = [the height of the posterior edge of fractured vertebral body/(the height of the posterior edge of caudal vertebral body + the height of the posterior edge of the cephalad vertebral body)/2] × 100%. Range and percentage of spinal shortening in a single spinal motion segment (total height of the posterior edge of vertebral body + cephalad disc + caudal disc) were calculated using the following equation on the postoperative lateral radiograph (Figure 4), range of spinal shortening: *X* = (*A* + *B*)/2 + *C* + *D*−E, percentage of spinal shortening of single spinal motion segment: *Y* = *X*/[(*A* + *B*)/2 + *C* + *D*] × 100%, in which A is the height of the posterior edge of the cephalad vertebral body, B is the height of the posterior edge of the caudal vertebral body, C is the height of the posterior edge of the cephalad disc, *D* is the height of the posterior edge of the caudal disc, and *E* is the height of the posterior edge of titanium mesh.

### 2.5. Statistical Analysis

Statistical analysis was performed by SPSS software (SPSS 17.0, IBM Corp, Armonk, NY). All quantitative data conformed to normal distribution by Kolmogorov–Smirnov test (*P* > 0.05). Therefore, the statistical description of the quantitative data was carried out by mean and standard deviation. The statistical description of the categorical data and ordinal data was carried out by number. For the further data analysis, methods including the *t*-test, the Pearson chi-square test, or Fisher exact test and Mann–Whitney *U* test were utilized, in which *P* < 0.05 represented the statistical significance.

## 3. Results

A total of 66 eligible patient cases were included in this retrospective case-control observational study, in which 32 patients received simple posterior decompression and fixation that were assigned to group A, and 34 patients received PIVCR and spinal shortening that was allocated to group B. There was no significant difference in two groups regarding age, gender, injury segment, and the time from their initial injury to prior to surgery (Table 1).

Compared with those in group A, the blood loss and average operative time were significantly more and longer in group B. The height of the posterior edge of the vertebral body was improved from 91.40 ± 1.04% preoperative to 95.16 ± 1.88% postoperative, 94.32 ± 1.61% at final follow-up in group A, while 91.88 ± 1.23%, 91.74 ± 7.03, and 91.70 ± 6.79%, respectively, in group B. The range of spinal shortening in group B was an average 1.57 ± 0.40 cm (range, 0.9 cm-2.28 cm) and 36.45 ± 6.56% of the height of the single spinal motion segment. Group B achieved better spinal shortening than group A (*P* < 0.05). The whole injured vertebral column and cephalad and the caudal intervertebral disc were removed in group B. Dural sacs were completely 360° decompressed, with significantly better decompression (*P* < 0.05) than group A (Table 2).

There was no neurological deterioration after surgery in any patient of the two groups. There was no significant difference in preoperative neurological impairment between the two groups (*P* > 0.05). At the final follow-up, the neurological improvement of patients in group B was 1.53 grades on average with the evaluation of ASIA impairment scale, which was significantly better than group A with 0.78 grades (*P* < 0.05). 89.47% of patients with incomplete deficit(ASIA B/C)in group B achieved more than 1 grade on the ASIA impairment scale, with mean recovery of around 1.79 grades. Besides, 60% of patients with a complete deficit(ASIA A)in group B achieved more than 1 grade on the ASIA impairment scale, with a mean recovery of around 1.2 grades. 62.5% of patients with an incomplete deficit in group A achieved more than 1 grade on the ASIA impairment scale, with a mean recovery of around 1 grade. 31.25% of patients with complete deficit achieved more than 1 grade on the ASIA impairment scale, with mean recovery of around 0.56 grades (Table 3).

Local back pain was significantly relieved in all patients after the operation. At the final follow-up, the VAS scores in group A were lower than in group B (*P* < 0.05). There was no difference between the preoperative and postoperative segmental Cobb angle groups. However, the lost segmental Cobb angle in group A was more significant than in group B at the final follow-up (*P* < 0.05) (Table 4). For the complications, one patient in group A and one in group B encountered superficial tissue infection that healed following debridement. Three patients in group A and two patients in group B suffered from urinary tract infections. One patient in group B experienced pneumonia. All respiratory and urinary complications got cured within 2 weeks after the operation.

## 4. Discussion

Previous literature demonstrated that early surgical treatment, including anterior, posterior, or anterior combined with posterior approaches, and pharmacological therapy were performed to treat thoracolumbar with SCI and obtained advanced achievements [4–8]. However, neurological improvement is not always ideal after the surgery. The neurological recovery in patients with severe traumatic SCI is particularly limited, especially in those with complete deficits [9–11]. Thus, controversy on the surgical technique treatment is still existed in various thoracolumbar fractures associated with sSCI [4, 5, 11]. Moreover, doubt has also been raised regarding pharmacological therapy in some research, which does not recommend the administration of GM-1 ganglioside and methylprednisolone for the management of acute SCI [26].

The primary traumatic injury resulting from traumatic mechanical injury to the spinal cord could disrupt the dynamics of SCBF and local ischemia-reperfusion, which may result in a further inflammatory response and bring about additional severe neurological injury [8, 27]. The severity of SCI is correlated with the degree of post-traumatic ischemia [28]. A large number of studies on SCI have shown that SCBF is significantly reduced after injury, which could lead to neurological deficits [16, 27, 29]. Werndle et al. and Gallagher et al. also confirm that the intraspinal pressure at the injury site is higher than subdural pressure below the injury or extradural pressure in traumatic SCI patients. Average intraspinal pressure from the patients with traumatic SCI is significantly higher than those without traumatic spinal cord injury, and the higher spinal cord perfusion pressure is correlated with increasing limb motor score [13, 14]. Therefore, surgery procedures including circumferential decompression, direct reduction of the tension in the spinal cord, and improvement of SCBF are all critical for neurological improvement in traumatic SCI patients.

In this study, 89.47% of patients with incomplete deficits underwent PIVCR and spinal shortening. At the final follow-up, patients achieved more than 1 grade on the ASIA scale with a mean recovery of around 1.79 grades. Besides, 60% of patients with complete deficit underwent PIVCR and spinal shortening, which achieved more than 1 grade on the ASIA scale with a mean recovery of around 1.2 grades at the final follow-up. However, 62.5% of patients with incomplete deficit underwent simple posterior decompression and fixation, achieving more than 1 grade on the ASIA impairment scale, with a mean recovery of around 1 grade. Only 31.25% of patients with complete deficit achieved more than 1 grade on the ASIA impairment scale, with a mean recovery of around 0.56 grades. The patients who underwent PIVCR and spinal shortening showed better clinical outcomes in neurological improvement than those who underwent simple posterior decompression and fixation surgery. Compared with the results reported by Harrop et al., Allain et al., and Lin et al., the recovery of neurological function was also significantly better in the group of PIVCR and spinal shortening [9–11]. PIVCR and spinal shortening may be important for neurological recovery in traumatic SCI. It could provide a new choice for the treatment of thoracolumbar fracture associated with sSCI.

Spine-shortening osteotomy has been a new procedure for the treatment of tethered cord syndrome with efficient reduction of neural tension and improvement of neurological deficit [21, 22]. PVCR and spinal shortening could reduce the spinal cord tension, which could not only achieve the correction rate of spinal deformity safely and effectively but also reduce the risk of neurological deficit secondary to operative procedures and indeed improve neurological deficits [17–19]. Jarvis et al. also found that spinal shortening could provide the effect of spinal cord decompression, improvement in spinal cord perfusion, and improvement in motor-evoked potentials (MEPs) when a reduction of MEP amplitude of greater than 50% occurred in severe spinal deformity correction [20]. The reperfusion of SCBF is of paramount importance in the improvement of spinal cord function after traumatic SCI. Proper range of spinal shortening brings about vasodilation of the arteries of the spinal cord, which could result in the concomitant reduction of resistance to blood flow and increase the diameter of the anterior spinal artery by 138% compared to base line. Moreover, SCBF could be increased by 111%-160% compared to the base line before shortening [19, 23, 25]. Based on the abovementioned studies, the spinal shortening could reduce the spinal tension and increase SCBF, which are critical for the neurological recovery in sSCI. Advantages including single posterior incision, circumferential decompression, correction in segmental kyphosis, direct reduction in the tension of the spinal cord, improvement of SCBF, intraspinal circumstances, and three-dimensional reconstruction could be brought about by PIVCR and spinal shortening. It could be hypothesized that the function of the remaining corticospinal tract may be waked up, and then it may create favorable conditions for the recovery of neurological function in patients with traumatic SCI in our study.

PIVCR is one of the corpectomy procedures to treat spinal deformity, which was first reported by Suk et al. in 2002 [30]. In the past decades, an increasing number of spinal deformity surgeons have used PIVCR to treat severe, rigid, and angular spinal deformity [31]. We first apply PIVCR procedure to treat thoracolumbar fracture associated with sSCI. The principle of PIVCR procedure in thoracolumbar fractures is appropriate spinal shortening. The total injured vertebrae with adjacent disks are resected completely to achieve 360° circumferential spinal cord decompression. Moreover, the space created by complete resection of vertebrae enables appropriate spinal shortening, reduction of spinal cord tension, and improvement of SCBF. The principle of spinal shortening was that the dural sacs should not be obvious shrinking and buckling. The main criterion to apply the PIVCR and spinal shortening procedure lies in no spinal cord complete transection at preoperative MRI, severe neurological deficit graded as ASIA A/B/C on the admission. PIVCR and spinal shortening are complex and risky techniques and can pose a challenge to surgeons. The risk of this procedure is that the surgical trauma is extensive and the intraoperative bleeding is massive compared to simple posterior decompression and fixation, while it could achieve circumferential decompression, better neurological recovery, less segmental Cobb loss, and fewer surgical complications. The non-neurological complications of PVCR include the following: a. respiratory system-related complications: pneumonia, pleural effusion, pulmonary embolism, pleural injury, hemopneumothorax, and respiratory failure; b. wound infection; c. excessive bleeding and traumatic coagulopathy; d cardiovascular complications: cardiac arrest, heart failure, arrhythmia, hypotension, and myocardial infarction; and e. deep vein thrombosis [31]. In this study, the complications encountered were including superficial infection, urinary tract infections, and pneumonia.

Some previous studies investigated the safe shortening range by building shortening of spine animal models and clinical experience. Kawahara et al. have confirmed that spinal shortening within 1/3 of the vertebral segment is the safe range which does not result in dural sac deformity. The warning range of spinal shortening could be considered as between 1/3 and 2/3 of the vertebral segment with shrinking and buckling of the dural sac but no spinal cord deformity. The spinal shortening of more than 2/3 of the vertebral segment is a dangerous range that could result in compressing the spinal cord by buckled dura and spinal cord deformity [23]. Ji et al. demonstrate that shortening of 1/2 of a vertebral segment height is safe, while spinal shortening between 1/2 and 2/3 of a vertebral segment may lead to incomplete SCI in canines [25]. Lu et al. report that the tolerance of the spinal cord can be increased and spinal cord injury resulting from angulation can be avoided when the spinal cord is shortened by 1/4 to 2/4 [32]. According to the study by Modi et al., the maximum safe range of spinal shortening in the pig is much to 104.2% of one vertebral body height [24]. However, the conclusion drawn by Modi et al. is based on increasing the length in laminectomy before spinal shortening. A review including seven clinical articles confirms that spinal shortening of 20.5 mm (range, 14 mm-25 mm) is the safe and effective surgical approach for treating tethered cord syndrome in humans [22]. We firmly oppose shortening the whole resection gap in incomplete neurological deficit patients, which may lead to excessive shrinking and warping of the dural sac and neurological deficit. In our study, the spinal shortening range is 1.57 cm and 36.45% of the single spinal motion segment. The range of spinal shortening in our study is similar to the safe range of spinal shortening in the literature reported previously.

There are several limitations to the clinical outcomes of this study due to the characteristics of a retrospective study, especially the limited number of patients and the clinical outcomes which need to be increased for further study. Future clinical prospective studies with larger groups of patients should apply randomization, controlled, and blind approaches in multicenter. Moreover, further study will directly measure the changes in spinal cord tension and SCBF to illuminate its therapeutic mechanism on neurological recovery.

## 5. Conclusions

PIVCR and spinal shortening is a safe, reliable, and effective surgical method for the treatment of sSCI-related thoracolumbar fractures.

## Figures and Tables

**Table 1:** Characteristics of patients in simple posterior decompression and fixation group (group A, *n* = 32) and PIVCR and spinal shortening group (group B, *n* = 34).

Patients characteristic	Surgical approach		
	Group A	Group B	*P*
Median age (years)	37.28 ± 13.40	34.00 ± 11.43	0.288
Sex (M/F)	19/13	22/12	0.655
Duration from initial injury to prior to surgery	17.44 ± 4.23	18.26 ± 3.20	0.372
TLICS scores	7.41 ± 1.19	7.65 ± 1.04	0.384
The sites of injured vertebral segment			0.905
T10	2	3	
T11	4	5	
T12	13	11	
L1	13	15	

**Table 2:** Parameters of patients in simple posterior decompression and fixation group (group A, *n* = 32) and PIVCR and spinal shortening group (group B, *n* = 34).

Parameter	Group A	Group B	*P*
Operative time(min)	179.53 ± 30.12	264.26 ± 48.39	<0.001^*∗*^
Blood loss (mL)	435.63 ± 222.61	564.41 ± 206.78	0.018^*∗*^
Spinal canal encroachment rate			
Preop.	57.80 ± 9.90	59.50 ± 10.20	0.496
Postop.	14.30 ± 4.03	0	<0.001^*∗*^
Heights of posterior edge of vertebral body (%)			
Preop.	91.40 ± 1.04	91.88 ± 1.23	0.614
Postop.	95.16 ± 1.88	91.74 ± 7.03	<0.001^*∗*^
Final follow-up	94.32 ± 1.61	91.70 ± 6.79	<0.001^*∗*^
Range of spinal shortening postop. (cm)	0.06 ± 0.03	1.57 ± 0.40	<0.001^*∗*^
Percentage of spinal shortening of single spinal motion segment (%)	1.47 ± 0.57	36.45 ± 6.56	<0.001^*∗*^

^*∗*^indicated statistically significant. Preop indicates preoperative; postop, postoperative.

**Table 3:** Neurological status of patients in simple posterior decompression and fixation group and PIVCR and spinal shortening group.

ASIA impairment scale on admission	Group A (*n* = 32)					Group B (*n* = 34)				
	A	B	C	D	E	A	B	C	D	E
A	11	2	2	1	0	6	5	1	1	2
B		5	2	1	0		2	2	3	3
C			1	2	5				1	8
Total	11	7	5	4	5	6	7	3	5	13

**Table 4:** Radiographic and clinic outcomes in simple posterior decompression and fixation group and PIVCR and spinal shortening group.

	Segmental kyphosis Cobb			VAS		
	Preop.	Postop.	At final follow-up	Preop.	Postop.	At final follow-up
Group A	24.84 ± 8.00	8.47 ± 2.82	11.19 ± 3.37	7.38 ± 1.24	3.34 ± 0.90	2.22 ± 1.34
Group B	26.85 ± 8.03	7.21 ± 3.38	8.26 ± 3.20	7.65 ± 1.07	3.06 ± 0.98	1.76 ± 1.02
*P*	0.313	0.105	0.001^*∗*^	0.342	0.225	0.028^*∗*^

^*∗*^indicated statistically significant. Preop indicates preoperative; postop, postoperative; VAS, visual analog scale.

## Data Availability

The datasets used and analyzed during the current study are available from the corresponding author upon reasonable request.

## Abbreviations

PIVCR: Posterior injured vertebra column resection
PVCR: Posterior vertebra column resection
sSCI: Severe spinal cord injury
SCBF: Spinal cord blood flow
MRI: Magnetic resonance imaging
ASIA: American Spinal Injury Association
VAS: Visual analog scale
MEP: Motor-evoked potentials.
